# A record-linkage study of post-stroke primary care psychological therapy effectiveness in England

**DOI:** 10.1038/s44220-025-00429-z

**Published:** 2025-06-05

**Authors:** Jae Won Suh, Vaughan Bell, Joshua E. J. Buckman, Céline El Baou, Roopal Desai, Caroline Fearn, Natalie L. Marchant, Marcus Richards, Claudia Cooper, Stephen Pilling, Amber John, Joshua Stott, Rob Saunders

**Affiliations:** 1 CORE Data Lab, Centre for Outcomes Research and Effectiveness, Research Department of Clinical, Educational and Health Psychology, UCL, London, UK; 2 Clinical, Educational and Health Psychology, UCL, London, UK; 3 Department of Neuropsychiatry, South London and Maudsley NHS Foundation Trust, London, UK; 4 Camden and Islington NHS Talking Therapies for anxiety and depression Services, North London NHS Foundation Trust, London, UK; 5 Adapt Lab, Research Department of Clinical, Educational and Health Psychology, UCL, London, UK; 6 Division of Psychiatry, UCL, London, UK; 7 MRC Unit for Lifelong Health and Ageing at UCL, UCL, London, UK; 8 Centre for Psychiatry and Mental Health, Wolfson Institute of Population Health, Queen Mary University London, London, UK; 9 North London NHS Foundation Trust, London, UK; 10 Department of Psychology, University of Liverpool, Liverpool, UK

**Keywords:** Health services, Stroke, Depression

## Abstract

At least one-third of stroke survivors are affected by depression or anxiety, but no large-scale studies of real-world clinical practice have assessed whether psychological therapies are beneficial for these patients. Here we show that psychological treatment is effective for stroke survivors on average, using national healthcare records from National Health Service Talking Therapies services in England, including 7,597 patients with a hospital diagnosis of stroke before attendance. Following psychological treatment, stroke survivors experienced moderate reductions in depression and large reductions in anxiety symptoms. Patients who started attending the services a year or more after a stroke were less likely to reliably recover from symptoms of depression or anxiety than those seen within six months of a stroke, irrespective of differences in baseline characteristics including age, gender, local area deprivation and symptom severity. Compared with a matched sample of patients without a stroke, stroke survivors were less likely to reliably recover and more likely to reliably deteriorate after psychological treatment, although adjusting for level of physical comorbidity attenuated these relationships. It is crucial that clinicians working with stroke survivors screen for symptoms of depression and anxiety and consider referring patients to primary care psychological therapies as early as possible.

## Abstract (web summary)

This study investigates the effectiveness of psychological therapies for depression and anxiety symptoms in 7,597 stroke survivors within England’s primary care system. Results show that 71.3% of stroke survivors experienced reliable improvement and significant reductions in symptom scores, underscoring the importance of intervention for improving recovery.

## Main

Stroke is the second leading cause of death and the third leading cause of disability worldwide^1^. There are approximately 1.3 million stroke survivors in the United Kingdom^2^, and this number is projected to more than double by 2035, partly due to the ageing population, better survival from stroke and increasing prevalence of risk factors^3^. It is estimated that depression affects one in three stroke survivors^4^ and can persist for years without intervention^5^, while anxiety affects more than one in four stroke survivors^6,7^. In the first year after stroke, people who had an ischemic stroke and intracerebral hemorrhage were two and four times more likely than the general population to have an incident depressive or anxiety disorder, respectively^8^. Furthermore, post-stroke depression or anxiety have been associated with impaired functional recovery from stroke (including physical impairment and cognitive function), higher mortality, lower quality of life and higher healthcare use^7,9^.

It is thus crucial to detect and treat common mental disorders (CMDs) such as depression and anxiety in stroke survivors to improve long-term health outcomes, cut demand on services and reduce healthcare costs. Current UK guidelines for stroke rehabilitation recommend that psychological therapies tailored to individual needs should be offered to stroke survivors^10,11^. Cognitive behavioral therapy is effective for reducing symptoms of depression and anxiety after stroke, but the sample sizes of the trials to date have been small (ranging from 5 to 322)^12,13^, and the robustness and generalizability of the findings to routine clinical practice remain uncertain^14^.

No study to our knowledge has investigated the effectiveness of psychological therapy routinely delivered in primary care for people who have had a stroke, even though the majority of psychological interventions are provided in these settings^15^. Despite the paucity of evidence, national stroke guidelines have recommended primary care interventions for the treatment of mild to moderate post-stroke depression and anxiety^16,17^. In addition, national guidelines for stroke rehabilitation from the National Institute of Health and Care Excellence (NICE) in England^10^ recommend that new or persisting emotional difficulties are assessed and considered for referral at reviews between 6 and 12 months after a stroke. However, the extent to which this is an optimal point for psychological therapy has not been tested.

The purpose of this study is to assess the effectiveness of psychological treatment for CMDs in stroke survivors across a nationally provided primary care psychological therapy program in England. The specific aims of this study were the following.

1. Examine the effectiveness of routinely delivered psychological therapies for reducing symptoms of depression or anxiety in stroke patients.
2. Assess whether treatment outcomes differed according to how soon after the stroke patients underwent psychological therapy.
3. Investigate whether psychological therapy outcomes differed between stroke survivors and people without an identified stroke.

## Study cohort

The study cohort comprised all adults aged ≥18 years who completed a course of psychological treatment in National Health Service (NHS) Talking Therapies for anxiety and depression (TTad) services between 2012 and 2019, and had a linked record in Hospital Episode Statistics (HES), including inpatient and outpatient records and associated diagnostic codes^18^, the Mental Health Services Dataset (MHSDS)^19^ and HES-ONS (Office of National Statistics) mortality data^20^ (see Methods and Supplementary Information A for further information on data sources). If participants had more than one episode of treatment in an NHS TTad service, only data from the first episode were used. A standard set of criteria used in studies of NHS TTad samples^21^ was used to exclude those who (1) had fewer than two sessions of psychological therapy, (2) did not meet the clinical threshold for depression (scored <10 on the Patient Health Questionnaire 9-item (PHQ-9)^22^) or generalized anxiety disorder (scored <8 on Generalized Anxiety Disorder Scale 7-item (GAD-7)^23^) or relevant Anxiety Disorder Specific Measure (Supplementary Information B), (3) had a primary diagnosis for which there is no evidence-based psychological therapy offered in NHS TTad (such as schizophrenia, bipolar disorder, alcohol dependence, bereavement) or (4) were still undergoing treatment in the available episode records. Patients missing data on baseline or follow-up measures on the PHQ-9 or GAD-7 were also excluded, but accounted for <1.5% of patients who received two or more sessions of treatment. For this study, participants who had a record of stroke during or after their first NHS TTad treatment were excluded. Of a total of 2,512,708 patients who attended ≥2 treatment sessions in NHS TTad between 2012 and 2019, 1,939,007 patients were included in the analyses, of whom 7,597 (0.4%) had a diagnosis of stroke before being assessed through NHS TTad (see Supplementary Information C for study flowchart).

## Results

### Baseline characteristics

Demographic characteristics and NHS TTad treatment-related variables of adults who had a stroke diagnosis and those without a stroke diagnosis before attending NHS TTad services are presented in Table 1. Compared with adults without a stroke diagnosis, adults who had a stroke were substantially older at referral to psychological treatment (mean age 57.8 versus 40.3 years), were more likely to be male and had a lower average GAD-7 score at assessment, and depression was more likely to be their presenting complaint (37.7% versus 28.8%). There was no difference in mean PHQ-9 scores between the two groups at assessment. Those with a stroke diagnosis were also more likely to report taking psychotropic medication and having at least one long-term physical health condition. In terms of treatment variables, those with a stroke diagnosis were more likely to have received treatment in recent years and had slightly fewer treatment sessions and a shorter waiting time between referral and assessment, but no difference in secondary waiting time (between assessment and treatment), compared with those without a stroke. Adults with a stroke diagnosis were more likely to complete NHS TTad treatment, but also more likely to be referred on or to be discharged, it having been assessed that NHS TTad services were not suitable for them. The likelihood of dropout from therapy was similar between those with and without a stroke diagnosis.

**Table 1:** Baseline characteristics in adults with and without a stroke diagnosis before attending NHS TTad services

	Stroke diagnosis	No diagnosis of stroke
	*N* = 7,597	*N* = 1,931,410
*Demographics*		
Age at referral—mean (s.d.)	57.8 (14.3)	40.3 (14.7)
	**% (*n*)**	**% (*n*)**
Age category		
18–24	1.5 (117)	15.9 (307,776)
25–44	15.0 (1,138)	46.4 (896,951)
45–64	50.9 (3,865)	31.1 (600,700)
65+	32.6 (2,477)	6.5 (125,983)
Ethnicity		
White	82.0 (6,227)	82.0 (1,582,968)
Mixed	1.1 (81)	1.9 (37,445)
Asian	3.8 (287)	4.3 (82,519)
Black	2.8 (213)	2.5 (47,759)
Other	0.9 (68)	1.1 (20,842)
Missing	9.5 (721)	8.3 (159,877)
Gender		
Male	47.6 (3,619)	32.8 (632,592)
Female	52.1 (3,956)	66.9 (1,292,057)
Missing	0.3 (22)	0.4 (6,761)
IMD quintile		
1 (most deprived)	22.3 (1,694)	21.6 (416,317)
2	20.8 (1,580)	21.3 (411,795)
3	19.8 (1,506)	19.5 (376,800)
4	17.7 (1,348)	17.9 (346,538)
5 (least deprived)	16.7 (1,271)	16.4 (316,141)
Missing	2.6 (198)	3.3 (63,819)
Employment status		
Employed	64.4 (4,891)	73.3 (1,415,202)
Unemployed	30.1 (2,285)	20.8 (401,619)
Missing/preferred not to answer	5.5 (421)	5.9 (114,589)
*Clinical measures pretreatment, pre-existing conditions and medication*		
	**Mean (s.d.)**	**Mean (s.d.)**
Depression symptoms pretreatment (PHQ-9)	15.8 (5.6)	15.8 (5.6)
Anxiety symptoms pretreatment (GAD-7)	13.4 (4.9)	14.3 (4.4)
Social Functioning (WSAS) pretreatment, prorated	19.7 (10.5)	19.5 (9.5)
	**% (*n*)**	**% (*n*)**
Taking psychotropic medication		
Yes	49.6 (3,766)	47.3 (913,023)
No	41.4 (3,147)	43.6 (841,399)
Missing	9.0 (684)	9.2 (176,988)
Self-reported long-term condition		
Yes	56.1 (4,262)	22.9 (441,637)
No	25.8 (1,959)	56.1 (1,083,073)
Missing	18.1 (1,376)	21.1 (406,700)
Record of hemiplegia/paraplegia		
Yes	22.5 (1,707)	0.43 (8,248)
No	77.5 (5,890)	99.6 (1,923,162)
*Treatment factors*		
	**% (*n*)**	**% (*n*)**
Diagnosis category		
Depression	37.7 (2,865)	28.8 (556,401)
Anxiety disorders		
Mixed anxiety and depressive disorder	14.3 (1,085)	16.5 (318,932)
GAD	14.3 (1,089)	15.7 (303,543)
OCD	0.5 (38)	1.7 (32,885)
PTSD	2.5 (189)	2.9 (55,879)
Phobic anxiety & panic	4.7 (356)	6.4 (123,295)
Other anxiety disorder	0.3 (26)	0.3 (5,972)
Missing	25.7 (1,949)	27.7 (534,503)
Appointment year		
2012	1.2 (90)	3.1 (60,011)
2013	5.1 (388)	11.2 (216,778)
2014	11.0 (834)	15.0 (288,885)
2015	16.3 (1,241)	17.4 (335,270)
2016	18.5 (1,408)	17.4 (335,955)
2017	20.5 (1,554)	16.4 (317,243)
2018	22.3 (1,696)	16.1 (311,660)
2019	5.1 (386)	3.4 (65,608)
Reason for ending therapy		
Completed	57.7 (4,385)	49.3 (951,940)
Dropout	21.5 (1,637)	21.6 (417,030)
Service not suitable	1.5 (112)	0.9 (16,609)
Declined	2.9 (221)	2.6 (50,468)
Referred on	4.2 (322)	3.3 (63,262)
Missing	12.1 (920)	22.4 (432,101)
	**Mean (s.d.)**	**Mean (s.d.)**
Number of sessions^a^	6.1 (4.2)	6.5 (4.6)
Time between referral and assessment (weeks)^a^	3.0 (3.8)	3.2 (4.3)
Time between assessment and treatment (weeks)^a^	6.8 (7.0)	6.7 (7.1)

IMD, Index of Multiple Deprivation; OCD, obsessive compulsive disorder; PTSD, post-traumatic stress disorder.

^a^To reduce the influence of extreme values, variables were winsorized at the top 99th percentile.

Data from NHS Digital.

### Treatment outcomes among adults who had a stroke

Among the 7,597 stroke survivors who were experiencing symptoms of depression or anxiety at assessment, 71.3% (*n* = 5,403) met the criteria for reliable improvement (see Methods for outcome definitions), 49.2% (*n* = 3,723) for reliable recovery and 7.3% (*n* = 554) for reliable deterioration at the end of treatment (Table 2). Mean symptom scores for both depression and generalized anxiety reduced over the course of NHS TTad therapy. PHQ-9 scores changed from 15.8 (s.d. 5.6) at assessment to 9.3 (6.9) after treatment, indicating a pre–post moderate effect size in terms of decrease in depression symptoms (Cohen’s *d*_av_ = −0.56). GAD-7 scores reduced from 13.4 (4.9) at assessment to 7.9 (5.9) after treatment, indicating a large reduction in anxiety symptoms (Cohen’s *d*_av_ = −0.93). Work and Social Adjustment Scale (WSAS)^24^ scores decreased from 19.7 (10.5) at assessment to 13.2 (10.8) after treatment, indicating a moderate reduction in functional impairment after treatment (Cohen’s *d*_av_ = −0.61). The findings were similar when restricting to those who had experienced an ischemic stroke. Among people who had an intracerebral hemorrhage, reliable improvement (73.3%) and reliable recovery (52.3%) was slightly more common, and reliable deterioration (6.1%) was slightly less common than in those with any stroke or an ischemic stroke. However, patients with intracerebral hemorrhage were the smallest group, and therefore the estimates had relatively wide confidence intervals (CIs).

**Table 2:** Treatment outcomes and changes in clinical measures among adults with any type of stroke, ischemic stroke and intracerebral hemorrhage, respectively

	Type of stroke diagnosis		
	Any stroke (I60–I64)	Ischemic stroke (I63)	Intracerebral hemorrhage (I61)
	*N* = 7,597	*N* = 5,235	*N* = 1,047
*Primary outcomes*			
	**% (95% CI)**	**% (95% CI)**	**% (95% CI)**
Reliable improvement	71.3 (70.3, 72.3)	71.0 (69.7, 72.2)	73.3 (70.6, 76.0)
Reliable recovery	49.2 (48.1, 50.4)	49.7 (48.3, 51.1)	52.3 (49.2, 55.3)
Reliable deterioration	7.3 (6.8, 7.9)	7.3 (6.6, 8.1)	6.1 (4.7, 7.7)
*Secondary outcomes*			
	**Mean (s.d.)**	**Mean (s.d.)**	**Mean (s.d.)**
PHQ-9 baseline	15.8 (5.6)	15.8 (5.6)	15.4 (5.5)
PHQ-9 after treatment	9.3 (6.9)	9.3 (6.9)	8.7 (6.5)
PHQ-9 change	6.5 (6.6)	6.6 (6.6)	6.7 (6.4)
GAD-7 baseline	13.4 (4.9)	13.3 (4.9)	13.2 (4.8)
GAD-7 after treatment	7.9 (5.9)	7.8 (5.9)	7.6 (5.7)
GAD-7 change	5.5 (5.9)	5.5 (5.9)	5.7 (5.8)
WSAS baseline	19.7 (10.5)	19.7 (10.5)	19.4 (10.8)
WSAS after treatment	13.2 (10.8)	13.1 (10.8)	13.4 (10.9)
WSAS change	6.6 (11.0)	6.7 (10.9)	5.9 (11.4)

*N*, data available.

### Impact of time between stroke diagnosis and attendance

People who started attending NHS TTad less than six months since being diagnosed with a stroke (*n* = 2,145) were on average slightly younger and more likely to be female, live in less deprived areas and be employed and less likely to be taking psychotropic medication compared with those who attended 12 or more months after a stroke (Supplementary Table D1). There was an increasing trend year on year in the number of patients who were diagnosed with a stroke 24 or more months before attending psychological therapy.

People who started attending NHS TTad less than six months after their stroke diagnosis were more likely to reliably improve and reliably recover and less likely to reliably deteriorate than those who started attending psychological therapy ≥6 months after their stroke (Supplementary Table D2). People who started psychological therapy sooner (<6 months after stroke diagnosis) had lower PHQ-9 and WSAS scores at assessment and saw greater reductions in these symptom scores compared with those who started later (≥6 months after stroke diagnosis). People who started psychological treatment sooner also had higher baseline GAD-7 scores but lower post-treatment scores than people who commenced later.

After adjusting for potential confounding factors (see Methods for further details), differences in the likelihood of reliable improvement and reliable deterioration by time between stroke diagnosis and attending NHS TTad no longer remained statistically significant (Table 3). Even after adjustment, however, people who started attending NHS TTad services ≥12 months after receiving a stroke diagnosis had 20% lower odds of reliable recovery compared with those who started attending psychological therapy less than six months after having a stroke. Additionally, adjusting for hemiplegia or paraplegia as a proxy for post-stroke physical disability did not alter these findings.

**Table 3:** Logistic regression results for primary treatment outcomes by time between stroke diagnosis and assessment at NHS TTad services (restricted to people with a stroke diagnosis, *N* = 7,597)

	Unadjusted	Standard adjustment^a^	Additional adjustment for hemiplegia/paraplegia
	OR (95% CI)	OR (95% CI)	OR (95% CI)
*Reliable improvement*			
Time between stroke diagnosis and attending NHS TTad			
<6 months	Reference group	Reference group	Reference group
6–11 months	0.80 (0.69–0.92)	0.87 (0.74–1.02)	0.87 (0.74–1.02)
12–23 months	0.80 (0.70–0.93)	0.88 (0.75–1.03)	0.88 (0.75–1.03)
24 months+	0.79 (0.69–0.90)	0.86 (0.74–1.01)	0.87 (0.74–1.01)
*Reliable recovery*			
Time between stroke diagnosis and attending NHS TTad			
<6 months	Reference group	Reference group	Reference group
6–11 months	0.87 (0.76–0.99)	0.91 (0.78–1.05)	0.91 (0.79–1.05)
12–23 months	0.78 (0.69–0.89)	0.80 (0.69–0.92)	0.81 (0.70–0.94)
24 months+	0.81 (0.72–0.92)	0.82 (0.71–0.94)	0.83 (0.72–0.95)
*Reliable deterioration*			
Time between stroke diagnosis and attending NHS TTad			
<6 months	Reference group	Reference group	Reference group
6–11 months	1.16 (0.90–1.49)	1.07 (0.81–1.41)	1.06 (0.81–1.40)
12–23 months	1.17 (0.92–1.50)	1.07 (0.81–1.40)	1.06 (0.81–1.39)
24 months+	1.10 (0.87–1.39)	0.97 (0.74–1.27)	0.97 (0.74–1.26)

^a^ORs adjusted for gender, age, ethnicity, IMD quintile, baseline PHQ-9 and GAD-7 scores, long-term physical health condition status, psychotropic medication status, appointment year, number of sessions, diagnosis category and waiting times.

### Comparison with patients who did not have a stroke

Adults with a stroke diagnosis were matched with a control group without stroke on key participant characteristics (see Supplementary Information E for matching procedure). Of the 6,895 adults with a diagnosis of stroke who had complete data for all continuous variables in the matching algorithm, only one person was unable to be matched. The final matched sample included 6,894 adults with a stroke diagnosis and 6,759 adults without identified stroke. Baseline characteristics after propensity score matching are presented in Supplementary Table E2. After matching, sample characteristics were largely similar between the two groups, although differences remained for variables that were not used in the matching procedure.

Primary and secondary treatment outcomes were compared between adults with and without a stroke diagnosis in the matched sample (Table 4, model 1). Participants who had a stroke were equally likely to reliably improve as the control group without a stroke (odds ratio, OR = 0.94, 95% CI = 0.87; 1.01) but had a 10% lower likelihood of reliable recovery (OR = 0.90, 95% CI = 0.85; 0.97) and 16% higher likelihood of reliable deterioration (OR = 1.16, 95% CI = 1.02; 1.33). For secondary outcomes, significantly smaller reductions in PHQ-9 (mean difference = −0.42, 95% CI = −0.64; −0.20) and GAD-7 (mean difference = −0.23, 95% CI = −0.42; −0.03) scores were observed in participants with a stroke diagnosis compared with those without. However, there were no statistically significant differences in the change in total WSAS (mean difference = −0.18, 95% CI = −0.60; 0.23) scores between the groups (Table 4).

**Table 4:** Primary and secondary treatment outcomes in adults with a stroke diagnosis compared with those without a stroke in the matched sample

Primary outcomes	Reliable improvement					Reliable recovery					Reliable deterioration				
	*N*	OR	95% CI	*P*	ICC	*N*	OR	95% CI	*P*	ICC	*N*	OR	95% CI	*P*	ICC
Model 1: unadjusted	13,751	0.94	(0.87, 1.01)	0.09		13,708	0.90	(0.85, 0.97)	<0.001		13,698	1.16	(1.02, 1.33)	0.03	
Model 2: standard adjustment^a^	13,751	0.96	(0.88, 1.03)	0.25		13,708	0.90	(0.84, 0.97)	<0.001		13,645	1.16	(1.01, 1.33)	0.04	
Model 3: model 2 + CCI	13,751	0.99	(0.92, 1.08)	0.89		13,708	0.94	(0.88, 1.01)	0.115		13,645	1.09	(0.95, 1.25)	0.235	
Model 4: clustering by CCG	13,630	0.93	(0.86, 1.01)	0.07	0.004	13,587	0.90	(0.84, 0.97)	<0.001	0.011	13,577	1.18	(1.03, 1.35)	0.02	0.021
Model 5: clustering by CCG + standard adjustment	13,630	0.95	(0.88, 1.03)	0.198	0.005	13,587	0.90	(0.84, 0.97)	0.004	0.008	13,577	1.17	(1.02, 1.35)	0.02	0.015
Model 6: model 5 + CCI	13,630	0.99	(0.91, 1.07)	0.796	0.005	13,587	0.94	(0.87, 1.01)	0.103	0.008	13,577	1.10	(0.96, 1.27)	0.163	0.014
**Secondary outcomes**	**PHQ-9 change**					**GAD-7 change**					**WSAS change (prorated)**				
	***N***	***b***	**95% CI**	***P***	**ICC**	***N***	***b***	**95% CI**	***P***	**ICC**	***N***	***b***	**95% CI**	***P***	**ICC**
Model 1: unadjusted	13,788	−0.42	(−0.64, −0.20)	<0.001		13,788	−0.23	(−0.42, −0.03)	0.02		10,032	−0.18	(−0.59, 0.23)	0.40	
Model 2: standard adjustment^a^	13,788	−0.30	(−0.49, −0.10)	<0.001		13,788	−0.18	(−0.36, −0.01)	0.04		10,032	−0.08	(−0.49, 0.33)	0.71	
Model 3: model 2 + CCI	13,788	−0.14	(−0.35, 0.06)	0.160		13,788	−0.10	(−0.27, 0.08)	0.275		10,032	−0.04	(−0.46, 0.38)	0.857	
Model 4: clustering by CCG	13,667	−0.44	(−0.66, −0.22)	<0.001	0.006	13,667	−0.24	(−0.44, −0.42)	0.02	0.004	9,997	−0.18	(−0.60, 0.23)	0.38	0.012
Model 5: clustering by CCG + standard adjustment	13,667	−0.3	(−0.50, −0.10)	0.003	0.006	13,667	−0.19	(−0.36, −0.01)	0.04	0.004	9,997	−0.08	(−0.48, 0.32)	0.70	0.01
Model 6: model 5 + CCI	13,667	−0.15	(−0.35, 0.05)	0.153	0.006	13,667	−0.10	(−0.28, 0.07)	0.258	0.004	9,997	−0.03	(−0.45, 0.38)	0.873	0.01

^a^Standard adjustment for gender, age at referral, ethnicity, IMD decile, PHQ-9 score at assessment, GAD-7 score at assessment, long-term physical health condition case, appointment year, psychotropic medication status, waiting time between referral and assessment, waiting time between assessment and treatment initiation, number of treatment sessions and diagnosis category.

*P* values were derived from two-tailed Wald tests (logistic regression) and two-tailed *t*-tests (linear regression) of the regression coefficients; *P* < 0.05 was considered statistically significant.

After standard adjustment for covariates (that is, all variables used for matching plus number of treatment sessions and diagnosis category), results for the primary outcomes remained virtually identical, while adjusted mean differences in the secondary outcomes were attenuated (model 2). Additionally, adjusting for physical comorbidity (using the Royal College of Surgeons adapted Charlson Comorbidity Index (CCI) as described in Supplementary Information G) attenuated all associations, such that none of the differences in primary or secondary treatment outcomes between patients with and without a stroke remained statistically significant (model 3).

Including a random intercept to account for potential clustering effects by Clinical Commissioning Groups (CCGs; for a full list of CCGs see Supplementary Information F) did not substantially alter any of these associations, and from intraclass correlation coefficients (ICCs) it was found that differences between CCGs accounted for only 0.4–2.1% of the variation in all treatment outcomes (models 4–6).

## Discussion

### Findings in context

In this large study, including all adults who received treatment from NHS TTad services in 2012–2019, we identified 7,597 stroke survivors. Of these 71.3% reliably improved and 49.2% reliably recovered from depression and anxiety following psychological treatment. This was in line with the UK Government target for the general population that 50% of eligible referrals to NHS TTad services should move to recovery; our study found that 49% of stroke survivors achieved reliable recovery, a more conservative measure of treatment outcome compared with recovery^25^. On average, stroke survivors experienced moderate reductions in depression and functional impairment and large reductions in anxiety symptoms after psychological treatment. These findings were consistent across two major stroke subtypes (ischemic stroke and intracerebral hemorrhage). People who started attending NHS TTad 12 months or more after a stroke had 20% lower odds of reliable recovery from depression and anxiety compared with those seen within six months of having a stroke, independent of various demographic and treatment-related factors (including age, gender, area deprivation, self-reported long-term physical condition and baseline symptom scores).

These findings were consistent with previous (albeit small) clinical trials reporting that cognitive behavioral therapy and other non-pharmacological therapies are effective in the treatment of depression and anxiety post-stroke^12,26^. This triangulation of evidence is important given that several previous trials had excluded populations with cognitive impairment, despite the fact that, in practice, treatments are often offered to people experiencing mild cognitive impairments^27,28^. Our findings also corroborate that psychological therapies offered in primary care are effective in reducing symptoms of depression and anxiety in people who have had a stroke. Therefore, taken together with evidence from previous prevalence studies, clinical trials and qualitative studies on the emotional needs of stroke survivors in rehabilitation^29^, our study supports current UK clinical guidelines, which recommend that people who have had a stroke should receive psychological therapy such as through primary care NHS TTad services^10,16^. In this study, people with a stroke diagnosis were less likely to reliably recover and more likely to reliably deteriorate after psychological treatment when compared with people without a stroke diagnosis matched on available participant characteristics. However, these differences were no longer statistically significant when adjusting for an index of physical comorbidities other than stroke, implying that poorer treatment outcomes in stroke survivors may be not due to stroke-specific factors in those who accessed treatment but due to the general burden of physical comorbidities experienced by the patients. These findings therefore do not detract from the fact that routinely delivered psychological therapy is likely to be effective for treating depression and anxiety in stroke survivors. Instead, it highlights the need for psychological interventions with suitable adaptations made for people with comorbid long-term physical health conditions, for instance as addressed by the NHS TTad Implementation Guidance for people with long-term and physical health conditions and medically unexplained symptoms^30^ and the NHS TTad *Positive Practice Guide: Older People*^31^.

Previous studies have shown that people who drop out of NHS TTad treatment have worse treatment outcomes than those who complete therapy, and tend to experience higher symptom severity and socioeconomic deprivation at referral^32,33^. The similar rates of dropout between stroke survivors and non-stroke survivors in this study may suggest that dropout is not strongly linked with stroke-specific factors. Even so, efforts to increase engagement with therapy among stroke survivors are likely to further improve treatment outcomes in this underserved patient group.

### Strengths and limitations

This study examines the effectiveness of routinely delivered psychological therapy in a cohort of stroke survivors. Major strengths of this study include the large number of stroke survivors and the national coverage of the dataset, supporting the generalizability of findings. Stroke survivors were identified through linked healthcare records, which may not completely capture all eligible patients, but stroke is more likely to be reliably recorded in healthcare records than other less serious conditions, and previous studies support the validity of cardiovascular outcomes including stroke in linked HES data^34,35^.

It is important to consider that stroke survivors who received treatment in NHS TTad are not necessarily representative of all individuals who have a stroke and require psychological treatment. People with more severe neurological consequences of stroke may not have been referred because, for example, they require integrated psychiatric treatment from a multidisciplinary team rather than focused intervention from a psychotherapist alone. As a more general concern, certain groups are underrepresented in NHS TTad (for example, people of minoritized ethnic backgrounds)^36^, so stroke survivors of these backgrounds may have been underrepresented. Incomplete records or loss to follow-up may also affect the representativeness of the dataset and be a potential source of bias. Nevertheless, less than 1.5% of the initial sample of patients who received two or more sessions of treatment were excluded due to missing data on outcomes in this study (Supplementary Information C), and it is therefore unlikely that the estimated average treatment effectiveness would be substantially biased as a result.

As our study had limited measures of stroke severity and disability due to stroke, the extent to which earlier initiation of psychological treatment is associated with better outcome independent of severity warrants further investigation. For example, we did not have information on post-stroke cognitive disabilities and sensory deficits. It is possible that people less impaired by stroke, who had better potential for recovery from depression and anxiety, were more likely to be referred early or accepted for intervention. Nevertheless, adjusting for hemiplegia or paraplegia as a proxy for physical disability (which is also associated with post-stroke cognitive impairment) did not alter our finding that earlier initiation of therapy was associated with a higher likelihood of reliable recovery.

Another limitation is that it is unknown whether depression and anxiety symptoms had started before or after the stroke, and whether patients had received treatment for such conditions before having a stroke, although these factors may be associated with treatment outcomes from CMDs in primary care^37,38^. There was also little information on lifestyle and behavioral risk factors such as smoking, physical activity and obesity, which are associated with both stroke and mental health symptoms^39,40^. Finally, the modified CCI does not fully capture the burden of physical comorbidities and how the effects of a stroke may interact with the patients’ other comorbidities. Despite limitations in the available data, this large-scale study leveraging routinely collected data from all services in a national primary care psychological therapy program provides unique real-world evidence that psychological therapy is effective for reducing symptoms of anxiety and depression among stroke survivors.

### Implications

The current study strongly supports the effectiveness of primary care psychological therapy as a first-line treatment for common mental health disorders after a stroke. Our findings also demonstrate that the earlier patients initiate psychological treatment after a stroke the better their likelihood of reliable recovery, although the extent to which this is modified by stroke severity and type requires further investigation. It is crucial that general practitioners, alongside other clinicians working with stroke survivors, screen for symptoms of depression and anxiety and consider referring patients to primary care psychological therapies such as NHS TTad as early as possible. It is also important that NHS TTad clinicians are offered and receive more training on treating people with long-term conditions, including those with cognitive deficits or sensory loss, and higher physical comorbidity burden in general. Such investment is likely to benefit both mental and physical health outcomes. Further research is needed to identify the characteristics of stroke survivors for whom primary care psychological therapy was judged suitable, but who did not respond well to therapy, to develop adaptations for effective care.

## Methods

### Data sources

The dataset for the current analyses was provided as part of the MODIFY study^41,42^, and includes all patients referred to the NHS TTad (formerly known as Improving Access to Psychological Therapies (IAPT))^43^ across all CCG areas in England between 2012 and 2019. NHS TTad services are free at the point of access, and available across England via self- or physician referral. These services offer a variety of evidence-based psychological therapies for CMDs, both at low intensity (including computerized and clinician-facilitated self-help interventions, predominantly based on cognitive behavioral therapy approaches), and at high intensity for moderate-to-severe presentations or for those with disorders for which there is no NICE recommended low-intensity treatment (for example, OCD, social anxiety disorder, health or illness anxiety disorder and PTSD). High-intensity therapies include clinician-led cognitive behavioral therapy, counseling, interpersonal psychotherapy, behavioral couples’ therapy, and eye movement desensitization and reprocessing).

Treatment intensity and type are decided on the basis of NICE guidelines for the primary mental health problem that forms the focus of treatment, as well as symptom severity and the patient’s goals and choices. Whilst low-intensity treatment is offered where there is evidence of effectiveness on the basis of the clinical presentation, patients who do not benefit from low-intensity intervention can be offered a high-intensity therapy within the same episode of care (stepped up). Patients may also be stepped down from high- to low-intensity therapy on the basis of regular review sessions. Like much other research using NHS TTad data^21,44,45^, this study analyzed data from the first episode of care received by each patient rather than focusing on the specific types of treatment, with the aim of understanding the average effectiveness of the routine psychological treatment provided by NHS TTad.

Using a unique patient identification key provided by NHS Digital, each participant was linked across routinely collected NHS datasets: NHS TTad, HES (including inpatient and outpatient records and associated diagnostic codes)^18^, MHSDS^19^ and HES-ONS mortality data^20^. The complete dataset contains information on sociodemographic characteristics (for example, gender, age, ethnicity), psychological therapy received (for example, referral and assessment dates, treatment outcomes) and other healthcare-related factors (date of hospital admission and diagnoses, date and cause of death) for individual patients. See Supplementary Information A for further information.

Patients or the public were not involved in the design, or conduct, or reporting, or dissemination plans of our research.

### Outcomes

Primary treatment outcomes were based on nationally determined outcome indicators used by NHS TTad providers and in previous studies^21,43^.

- Reliable improvement: a reduction in depression or anxiety symptoms from the first to last attended treatment session that exceeds the threshold for error of measurement on the corresponding symptom scale (≥6 points on PHQ-9, ≥4 points on GAD-7; see Supplementary Information B for Anxiety Disorder Specific Measure thresholds).
- Reliable recovery: achieving both reliable improvement (as defined above) and ending treatment below the clinical threshold for ‘caseness’ on the measures of both depression and anxiety. Caseness refers to a level of symptoms likely to be sufficient to meet the diagnostic criteria for the measured disorder (≥10 on PHQ-9, ≥8 on GAD-7; see Supplementary Information B for Anxiety Disorder Specific Measure thresholds).
- Reliable deterioration: an increase in depression or anxiety symptoms from the first to last attended treatment session by at least the magnitude of the threshold for the error of measurement (see reliable improvement above for details).

Secondary outcomes were pre–post score changes on measures of depression (PHQ-9), generalized anxiety (GAD-7) and functional impairment (WSAS)^24^. Depression and anxiety measures were taken from the NHS TTad dataset. Data on WSAS were available for a subset of participants only, as they were measured and available nationally in NHS TTad only from 2015 onwards. For individuals not in employment, their total WSAS score was ‘prorated’, meaning thst the average value of the scores for all other items was imputed as the score for the item on ability to work. Further details on these measures are provided in Supplementary Information H.

### Exposure

Diagnosis of stroke before the date of assessment at NHS TTad was identified using ICD-10 (the International Classification of Diseases, 10th Revision) codes I60–I64 in the HES and MHSDS databases. Two major stroke subtypes were identified separately with the following codes: I61 (intracerebral hemorrhage) and I63 (ischemic stroke). The use of these codes to ascertain stroke in administrative databases such as HES has been validated^34,35,46^.

### Covariates

A range of demographic and clinical covariates potentially associated with therapy outcomes and with stroke were adjusted for in the analyses. Self-reported demographic information from NHS TTad data included gender, age at referral, ethnicity and IMD quintile (1, most deprived 20% of geographical areas in England; 5, least deprived 20%). Information on psychological treatment received and mental health were taken from NHS TTad data, which included diagnosis category (for example, depression, generalized anxiety disorder, OCD, PTSD), pretreatment PHQ-9 and GAD-7 scores, appointment year, number of treatment sessions attended, self-reported measures of psychotropic medication use and presence of a self-reported long-term physical health condition. Waiting times from referral to assessment, and assessment to treatment, in weeks, were calculated from appointment dates. Additionally, the burden of physical comorbidity at NHS TTad assessment was measured using the Royal College of Surgeons CCI^47^, adapted to exclude cerebrovascular disease and hemiplegia or paraplegia from the list of contributing conditions (Supplementary Information G).

### Statistical analysis

This consisted of the following analyses.

1. Summary statistics of demographic characteristics and treatment-related factors for adults with and without a diagnosis of stroke before therapy. Missing data for categorical variables were assigned a separate category.
2. Primary NHS TTad treatment outcomes (that is, reliable improvement, recovery, deterioration) reported separately by subsamples with stroke (I60–I64), ischemic stroke (I63) or intracerebral hemorrhage (I61) before NHS TTad assessment. Pre–post treatment differences in symptoms of depression, anxiety and functional impairment among stroke survivors were also reported. Effect sizes were calculated using the adapted Cohen *d* for within-subject design (Cohen’s *d*_av_)^48^.
3. Multiple logistic regression using time between stroke diagnosis and assessment at NHS TTad (<6 months (reference category), 6–12 months, 12–24 months and 24+ months) as the explanatory variable to assess the association with three primary NHS TTad treatment outcomes.
4. To investigate whether diagnosis of stroke is associated with treatment outcomes, adults with a stroke diagnosis were matched with control participants without identified stroke. A propensity score was estimated using logistic regression including factors associated with the outcomes as covariates (for example, gender, age at referral), and used to find the most appropriate control^49^. See Supplementary Information E for more details on the matching procedure.

Logistic and linear regressions were fitted for binary and continuous outcomes, respectively. Additional adjustment for covariates and multilevel analyses by CCGs were conducted to control for potential confounding due to patient characteristics, treatment-related factors and CCG-level variation. Whether additionally adjusting for the burden of physical comorbidity changed the associations between stroke diagnosis and treatment outcomes was also investigated using the CCI. Potential for multicollinearity among the covariates in the regression models was assessed by examining pairwise correlations, and no cause for concern was detected. Models were run in the following order.

- Model 1 (unadjusted): diagnosis of stroke as a single exposure.
- Model 2: model 1 with standard adjustments (for gender, age at referral, ethnicity, IMD decile, PHQ-9 score at assessment, GAD-7 score at assessment, long-term physical health condition case, appointment year, psychotropic medication status, waiting time between referral and assessment, waiting time between assessment and treatment initiation, number of treatment sessions and diagnosis category).
- Model 3: model 2 adjusted for CCI as a measure of physical comorbidity.
- Model 4: model 1 including a random intercept to consider potential clustering effects by NHS CCGs.
- Model 5: model 4 with standard adjustments.
- Model 6: model 5 adjusted for CCI.

All analyses were conducted in Stata 17^50^. This study followed the Enhancing the Quality and Transparency of Health Research reporting guideline “The REporting of studies Conducted using Observational Routinely-collected health Data (RECORD) statement”^51^.

### Ethics statement

All data sources were fully anonymized, and a linkage key was provided by NHS Digital, for records from each database to be linked at the individual patient level using an anonymized subject identifier. Non-identifiable information was provided by NHS Digital with a legal basis for the anonymization, meaning that this research did not require research ethics committee review, as per the Governance Arrangements of Research Ethics Committees.

### Reporting summary

Further information on research design is available in the Nature Portfolio Reporting Summary linked to this article.

## Supplementary information

Supplementary Information Supplementary information on data (A and B), study flowchart (C), baseline tables (Tables D1 and D2), information on matching procedure (Tables E1 and E2), information on CCGs (F), information on comorbidity index (G) and information on secondary outcome measures (H).

Reporting Summary

## Data Availability

This work uses data provided by patients and collected by the NHS as part of their care and support. All data used for this study are available upon successful application to NHS Digital via the Data Access Request Service (DARS): https://digital.nhs.uk/services/data-access-request-service-dars. Data fields can be accessed via the NHS Digital data dictionary: https://www.datadictionary.nhs.uk/. Further information on the datasets is available from the following links: https://digital.nhs.uk/data-and-information/data-collections-and-data-sets/data-sets/improving-access-to-psychological-therapies-data-set; https://digital.nhs.uk/services/hospital-episode-statistics; https://digital.nhs.uk/data-and-information/data-collections-and-data-sets/data-sets/mental-health-services-data-set; https://digital.nhs.uk/services/data-access-request-service-dars/dars-products-and-services/data-set-catalogue/civil-registrations-of-death.

## Code availability

The code used for statistical analyses in this study is publicly available on GitHub at https://github.com/jae-suh74/MODIFY_Stroke.
