# Supplementary material for: A record-linkage study of post-stroke primary care psychological therapy effectiveness in England
Source: Nat Ment Health. 2025 Jun 5;3(6):626–35. doi: 10.1038/s44220-025-00429-z (PMC12165844; doi:10.1038/s44220-025-00429-z)
Supplement: Supplementary file 1 — Supplementary information on data (A and B), study flowchart (C), baseline tables (Tables D1 and D2), information on matching procedure (Tables E1 and E2), information on CCGs (F), information on comorbidity index (G) and information on secondary outcome measures (H). [file 44220_2025_429_MOESM1_ESM.pdf]

nature mental health

Supplementary information

https://doi.org/10.1038/s44220-025-00429-z

A record-linkage study of post-stroke primary care psychological therapy effectiveness in England

In the format provided by the authors and unedited

Supplementary online materials to:

A record-linkage study of post-stroke primary care psychological therapy effectiveness in 1.9m adults in England

Contents

A) MODIFY Study dataset 2
B) Clinical thresholds for Anxiety Disorder Specific Measures (ADSMs) 3
C) Study flowchart 4
D) Comparison of treatment outcomes by time between stroke diagnosis and assessment in NHS TTad 5
E) Propensity score matching 9
F) Clinical commissioning group categories 14
G) Adjusting for the Charlson Comorbidity Index 17
H) Secondary outcome measures 19

A) MODIFY Study dataset

**NHS TTad dataset (1)**	Routinely collected data for every patient seen in NHS TTad services across all clinical commissioning group areas in England between 2012 to 2019. Includes demographic (e.g., gender, age, ethnicity), therapy (e.g., referral and assessment dates, treatment information at each appointment) and outcome (improvement, recovery, deterioration) information for individual patients.<br><br>NHS TTad services are free at the point of access, and available across England via self- or physician referral. These services offer a variety of evidence-based psychological therapies for CMDs, at both low intensity (including computerised and clinician facilitated self-help interventions, predominantly based on cognitive behavioural therapy [CBT] approaches), and high intensity for moderate-to-severe presentations or for those with disorders for which there is no NICE recommended low intensity treatment (e.g. obsessive compulsive disorder [OCD], social anxiety disorder, health or illness anxiety disorder, and posttraumatic stress disorder [PTSD]). High-intensity therapies include clinician-led CBT, counselling, and interpersonal psychotherapy (IPT), behavioural couples' therapy, and eye movement desensitisation and reprocessing [EMDR]).
**Hospital Episode Statistics dataset (2)**	Admitted Patient Care and Outpatient datasets from all National Health Service (NHS) hospitals across England. Includes demographic (e.g., age, ethnicity, gender), geographical (e.g., residential area, area treatment received), administrative (e.g., dates of admission and discharge), and clinical (e.g., diagnoses, treatments, operations) information for individual patients. Data were available to 31 March 2020.
**Mental Health Services dataset (3)**	Previously known as the Mental Health Minimum Dataset (MHMDS) and the Mental Health and Learning Disability Dataset (MHLDDS). Includes data from secondary care services (e.g., provided in hospitals, outpatient clinics, in the community) for mental illness, learning disability, autism, and other neurodevelopmental conditions. Data were available to 31 March 2019.
**HES-ONS Mortality dataset (4)**	Linked information from HES and Office of National Statistics (ONS) mortality data. Includes cause, date, and place of death (both in and out of hospital). Data were available to 1 June 2020.

B) Clinical thresholds for Anxiety Disorder Specific Measures (ADSMs)

Anxiety disorder	Recommended ADSM	Threshold for caseness	Threshold for reliable improvement
Agoraphobia	Mobility Inventory (5))	2.3	0.73
Health anxiety	Health Anxiety Inventory (6)	18	4
Obsessive compulsive disorder (OCD)	Obsessive Compulsive Inventory (7)	40	32
Panic disorder	Panic Disorder Severity Scale (8)	-	-
Post-traumatic stress disorder (PTSD)	Impact of Events Scale (IES-R) (9)	33	9
Social anxiety disorder	Social Phobia Inventory (10)	19	10

C) Study flowchart

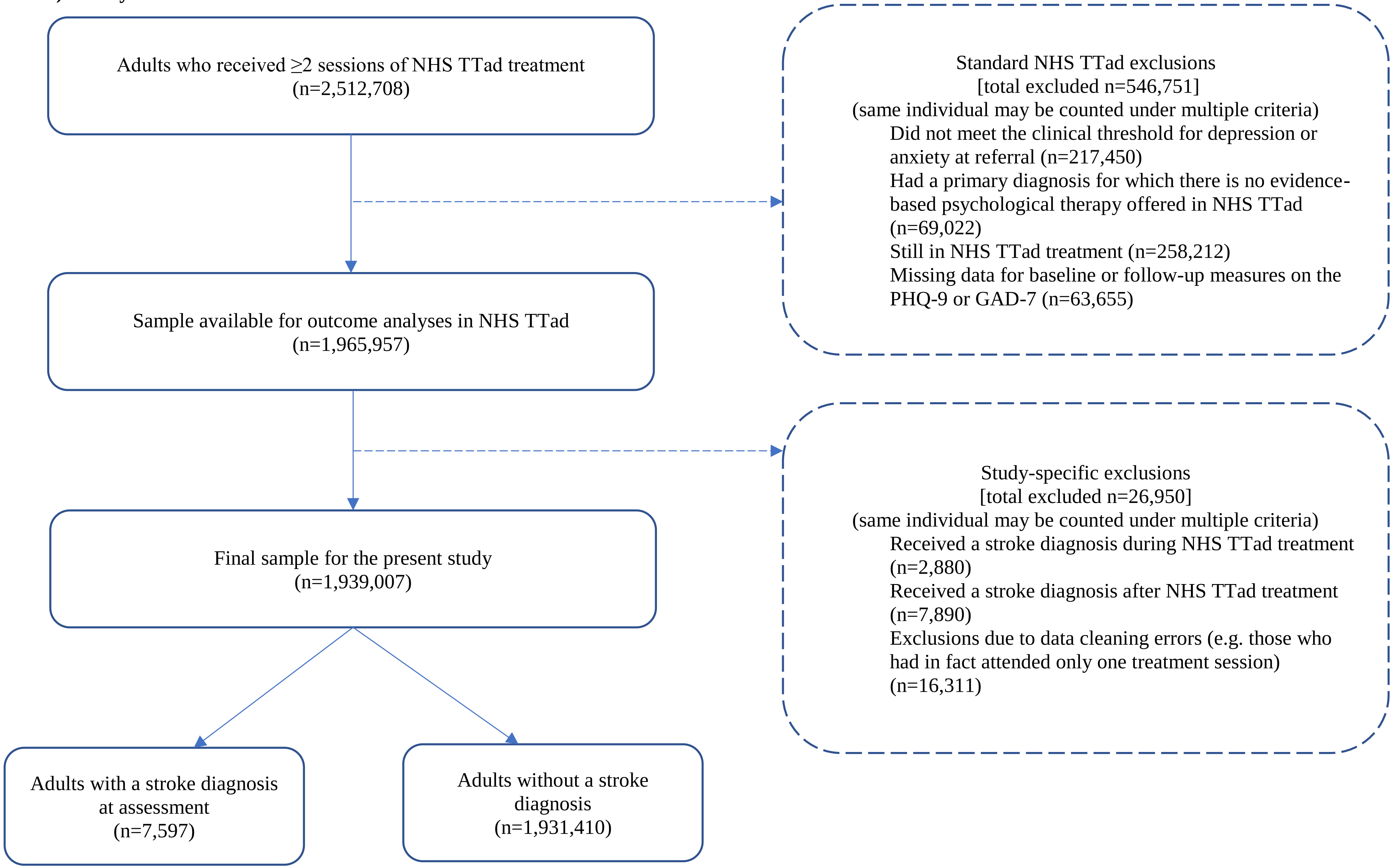

D) Comparison of treatment outcomes by time between stroke diagnosis and assessment in NHS TTad

Table D1. Baseline characteristics by time between stroke diagnosis and NHS TTad assessment

Demographic and baseline measures	Time between stroke diagnosis and NHS TTad assessment (months)				
	**<6 months**	**6-11 months**	**12-23 months**	**24 months +**	**No diagnosis of stroke**
	N=2,145	N=1,530	N=1,735	N=2,187	N=1,931,410
**DEMOGRAPHICS**					
Age at referral - Mean (SD)	57.2 (14.1)	58.7 (14.2)	57.5 (14.3)	58.1 (14.7)	40.3 (14.7)
	**% (n)**	**% (n)**	**% (n)**	**% (n)**	**% (n)**
**Age category**					
18-24	1.4 (31)	1.6 (25)	1.2 (21)	1.8 (40)	15.9 (307,776)
25-44	15.9 (342)	13.5 (206)	16.1 (279)	14.2 (311)	46.4 (896,951)
45-64	51.8 (1,111)	50.7 (776)	50.0 (868)	50.8 (1,110)	31.1 (600,700)
65+	30.8 (661)	34.2 (523)	32.7 (567)	33.2 (726)	6.5 (125,983)
**Ethnicity**					
White	82.1 (1,762)	80.3 (1,228)	82.4 (1,430)	82.6 (1,807)	82.0 (1,582,968)
Mixed	0.9 (19)	1.2 (19)	1.0 (18)	1.1 (25)	1.9 (37,445)
Asian	4.6 (99)	3.3 (50)	3.7 (64)	3.4 (74)	4.3 (82,519)
Black	2.6 (55)	3.7 (56)	2.2 (39)	2.9 (63)	2.5 (47,759)
Other	1.1 (23)	0.8 (12)	0.8 (14)	0.9 (19)	1.1 (20,842)
Missing	8.7 (187)	10.8 (165)	9.8 (170)	9.1 (199)	8.3 (159,877)
**Gender**					
Male	45.7 (980)	45.4 (695)	50.0 (868)	49.2 (1,076)	32.8 (632,592)
Female	54.1 (1,161)	54.2 (830)	49.7 (862)	50.4 (1,103)	66.9 (1,292,057)
Missing	0.2 (4)	0.3 (5)	0.3 (5)	0.4 (8)	0.4 (6,761)
**IMD quintile**					
1 (Most deprived)	20.8 (447)	21.6 (330)	22.5 (391)	24.1 (526)	21.6 (416,317)
2	20.5 (439)	20.8 (319)	20.1 (349)	21.6 (473)	21.3 (411,795)
3	19.7 (423)	20.8 (319)	18.6 (323)	20.2 (441)	19.5 (376,800)
4	17.9 (385)	17.6 (270)	18.4 (319)	17.1 (374)	17.9 (346,538)

5 (Least deprived)	18.1 (389)	15.8 (241)	17.4 (302)	15.5 (339)	16.4 (316,141)
Missing	2.9 (62)	3.3 (51)	2.9 (51)	1.6 (34)	3.3 (63,819)
**Employment status**					
Employed	70.7 (1,517)	65.0 (994)	61.1 (1,060)	60.4 (1,320)	73.3 (1,415,202)
Unemployed	24.5 (525)	29.0 (444)	32.8 (569)	34.2 (747)	20.8 (401,619)
Missing/preferred not to answer	4.8 (103)	6.0 (92)	6.1 (106)	5.5 (120)	5.9 (114,589)
**CLINICAL MEASURES PRE-TREATMENT, PRE-EXISTING CONDITIONS AND MEDICATION**					
	**Mean (SD)**	**Mean (SD)**	**Mean (SD)**	**Mean (SD)**	**Mean (SD)**
Depression symptoms pre-treatment (PHQ-9)	15.5 (5.5)	15.7 (5.5)	15.9 (5.6)	16.1 (5.6)	15.8 (5.6)
Anxiety symptoms pre-treatment (GAD-7)	13.8 (4.6)	13.2 (5.0)	13.4 (4.9)	13.3 (4.9)	14.3 (4.4)
Social Functioning (WSAS) pre-treatment, pro-rated	19.2 (10.4)	20.1 (10.7)	19.7 (10.4)	20.0 (10.4)	17.8 (8.9)
	**% (n)**	**% (n)**	**% (n)**	**% (n)**	**% (n)**
**Taking psychotropic medication**					
Yes	45.2 (970)	47.2 (722)	52.3 (907)	53.4 (1,167)	47.3 (913,023)
No	46.1 (989)	42.3 (647)	38.0 (659)	39.0 (852)	43.6 (841,399)
Missing	8.7 (186)	10.5 (161)	9.7 (169)	7.7 (168)	9.2 (176,988)
**Self-reported long-term condition**					
Yes	53.2 (1,142)	55.2 (844)	57.1 (991)	58.8 (1,285)	22.9 (441,637)
No	27.4 (588)	24.2 (371)	26.2 (454)	25.0 (546)	56.1 (1,083,073)
Missing	19.3 (415)	20.6 (315)	16.7 (290)	16.3 (356)	21.1 (406,700)
**TREATMENT FACTORS**					
**Diagnosis category**					
Depression	33.8 (725)	35.6 (545)	37.6 (653)	43.1 (942)	30.6 (556,401)
Anxiety disorders					
Mixed anxiety and depressive disorder	13.1 (281)	15.4 (236)	15.1 (262)	14.0 (306)	17.5 (318,932)
GAD	17.3 (372)	13.5 (206)	12.3 (213)	13.6 (298)	16.7 (303,543)
OCD	0.7 (14)	0.1 (2)	0.4 (7)	0.7 (15)	1.8 (32,885)
PTSD	1.7 (37)	2.7 (42)	2.7 (46)	2.9 (64)	3.1 (55,879)
Phobic anxiety & panic	4.8 (102)	4.4 (68)	4.1 (72)	5.2 (114)	6.8 (123,295)

Other anxiety disorder	0.3 (6)	0.3 (5)	0.5 (8)	0.3 (7)	0.3 (5,972)
Missing	28.3 (608)	27.8 (426)	27.3 (474)	20.2 (441)	23.3 (423,323)
**Year of treatment**					
2012	4.1 (88)	0.1 (2)	0.0 (0)	0.0 (0)	3.1 (60,011)
2013	10.3 (220)	8.5 (130)	2.1 (37)	0.0 (1)	11.2 (216,778)
2014	13.8 (295)	16.1 (247)	14.6 (254)	1.7 (38)	15.0 (288,885)
2015	16.4 (352)	17.6 (270)	20.6 (358)	11.9 (261)	17.4 (335,270)
2016	17.0 (365)	17.8 (272)	19.1 (331)	20.1 (440)	17.4 (335,955)
2017	18.0 (387)	15.8 (241)	19.2 (333)	27.1 (593)	16.4 (317,243)
2018	17.2 (368)	19.7 (301)	20.6 (358)	30.6 (669)	16.1 (311,660)
2019	3.3 (70)	4.4 (67)	3.7 (64)	8.5 (185)	3.4 (65,608)
**Reason for ending therapy**					
Completed	52.4 (1,125)	55.1 (843)	59.1 (1,026)	63.6 (1,391)	49.3 (951,940)
Dropout	19.7 (422)	19.8 (303)	22.1 (384)	24.1 (528)	21.6 (417,030)
Service not suitable	1.1 (24)	1.0 (15)	1.8 (31)	1.9 (42)	0.9 (16,609)
Declined	2.2 (47)	3.3 (50)	2.8 (49)	3.4 (75)	2.6 (50,468)
Referred on	3.7 (79)	4.1 (62)	4.6 (79)	4.7 (102)	3.3 (63,262)
Missing	20.9 (448)	16.8 (257)	9.6 (166)	2.2 (49)	22.4 (432,101)
	**Mean (SD)**	**Mean (SD)**	**Mean (SD)**	**Mean (SD)**	**Mean (SD)**
Number of sessions[a]	6.0 (4.2)	6.1 (4.3)	6.2 (4.3)	6.0 (4.0)	6.5 (4.6)
Time between referral and assessment (weeks)[a]	2.7 (3.2)	3.6 (4.3)	3.3 (4.3)	2.8 (3.3)	3.2 (4.3)
Time between assessment and treatment (weeks)[a]	6.6 (6.8)	6.5 (7.0)	6.8 (7.2)	7.1 (7.1)	6.7 (7.1)

Abbreviations: IMD = Index of multiple deprivation, GAD = Generalised anxiety disorder, OCD=Obsessive compulsive disorder, PTSD=Post traumatic stress disorder, PHQ=Patient health questionnaire, SD=Standard Deviation, HES=Hospital Episode Statistics

[a]To reduce the influence of extreme values, variables were winsorized at the top 99% percentile

Table D2. Clinical measures and outcomes by time between stroke diagnosis and NHS TTad assessment

	Time between stroke diagnosis and NHS TTad assessment (months)				
	**<6 months**	**6-11 months**	**12-23 months**	**24 months +**	**No diagnosis of stroke**
	N=2,145	N=1,530	N=1,735	N=2,187	N=1,931,410
**PRIMARY OUTCOMES**					
	**% (n)**	**% (n)**	**% (n)**	**% (n)**	**% (n)**
Reliable improvement	74.6 (1,595)	70.0 (1,069)	70.2 (1,214)	69.8 (1,525)	70.7 (1,359,657)
Reliable recovery	52.8 (1,126)	49.4 (752)	46.6 (806)	47.7 (1,039)	47.5 (910,442)
Deterioration	6.7 (143)	7.7 (117)	7.8 (134)	7.3 (160)	6.4 (122,656)
**SECONDARY OUTCOMES**					
	**Mean (SD)**	**Mean (SD)**	**Mean (SD)**	**Mean (SD)**	**Mean (SD)**
PHQ9-Baseline	15.5 (5.5)	15.7 (5.5)	15.9 (5.6)	16.1 (5.6)	15.8 (5.6)
PHQ9-After treatment	8.7 (6.6)	9.4 (6.9)	9.6 (6.9)	9.7 (7.0)	9.4 (6.8)
PHQ9 Change	6.8 (6.5)	6.3 (6.5)	6.3 (6.6)	6.4 (6.6)	6.4 (6.6)
GAD7-Baseline	13.8 (4.6)	13.2 (5.0)	13.4 (4.9)	13.3 (4.9)	14.3 (4.4)
GAD7-After treatment	7.8 (5.8)	8.0 (6.0)	8.1 (5.9)	8.0 (6.0)	8.5 (5.9)
GAD7 Change	6.0 (5.8)	5.3 (6.1)	5.3 (5.9)	5.3 (5.8)	5.9 (5.9)
WSAS-Baseline	19.2 (10.4)	20.1 (10.7)	19.7 (10.4)	20.0 (10.4)	19.5 (9.5)
WSAS-After treatment	12.4 (10.4)	13.6 (11.0)	13.3 (10.7)	13.5 (11.0)	12.9 (10.1)
WSAS Change	7.0 (11.0)	6.6 (11.2)	6.4 (11.0)	6.5 (10.8)	6.6 (10.1)

Abbreviations: n = Data available; SD = Standard Deviation; PHQ=Patient Health Questionnaire ;GAD=Generalised Anxiety Disorder; WSAS=Work and Social Activities Scale

E) Propensity score matching

Adults with a stroke diagnosis were matched with control participants without identified stroke using the *psmatch2* command in Stata (11). A propensity score was used to find the most appropriate control based on key covariates. It was estimated using logistic regression by modelling the probability to belong to the stroke group. The model included all factors thought to be associated with the outcomes. Baseline employment status, however, was not used for the matching procedure as it was conceptualised as a mediator in the relationship between stroke and depression and anxiety symptoms. Controlling for a mediator variable will result in artificially attenuated associations between the exposure and the outcome. A summary of the propensity score model is presented in Table E1.

Table E1. Propensity score estimation model, Logistic regression

	Coefficient	Standard Error	p-value
**Gender**			
Male (reference category)			
Female	-0.55147	0.024435	<.001
Missing	-0.54873	0.217328	0.012
**Age at referral**	0.064048	0.000838	<.001
**Ethnicity**			
White (reference category)			
Mixed	0.054997	0.117218	0.639
Asian	0.141578	0.066065	0.032
Black	0.345207	0.076585	<.001
Other	0.086582	0.12783	0.498
Missing	0.293396	0.045003	<.001
**IMD decile**			
1 (reference category)			
2	-0.03925	0.052265	0.453
3	-0.05783	0.052301	0.269
4	-0.1432	0.053199	0.007
5	-0.11938	0.053023	0.024
6	-0.15544	0.053743	0.004
7	-0.2076	0.054637	<.001
8	-0.26599	0.055763	<.001
9	-0.1946	0.055296	<.001
10	-0.26219	0.05735	<.001
Missing	-0.03756	0.084072	0.655
**PHQ-9 score at assessment**	0.018276	0.002616	<.001

	Coefficient	Standard Error	p-value
**GAD-7 score at assessment**	-0.02708	0.003053	<.001
**LTC case**			
No (reference category)			
Yes	1.102625	0.029656	<.001
Missing	0.418221	0.040107	<.001
**Appointment year**			
2012 (reference category)			
2013	0.945393	0.180301	<.001
2014	1.402476	0.175749	<.001
2015	1.652214	0.174474	<.001
2016	1.760916	0.174241	<.001
2017	1.873867	0.174116	<.001
2018	1.950261	0.174025	<.001
2019	2.014329	0.180157	<.001
**Psychotropic medication**			
Prescribed not taking (reference category)			
Prescribed and taking	0.091572	0.066157	0.166
Not prescribed	0.19688	0.066808	0.003
Missing	0.20922	0.076152	0.006
**Waiting time between referral and assessment**	0.004316	0.003077	0.161
**Waiting time between assessment and treatment initiation**	0.000756	0.001738	0.664
**Intercept**	-10.5183	0.199744	<.001

Model summary:

N Obs: 1,765,628

Likelihood ratio $\chi^2(34)$: 11796.90

Prob>Chi-square=<.001

Pseudo R^2=0.1307

Abbreviations: IMD = Index of multiple deprivation, GAD = Generalised anxiety disorder, PHQ=Patient health questionnaire, LTC=Long term condition

The quality of matching was assessed by comparing demographic characteristics of the group with stroke and control group without stroke before and after matching. Where a control was identified as an appropriate match

for more than one participant in the sample with stroke, these were weighted and used in the analysis (maximum weight = 3).

A matched control was identified for all but one observation out of the 6,895 individuals in the group with stroke with complete data on all continuous variables used for matching. 129 individuals in the control group were matched to two individuals in the stroke group. 3 individuals in the control group were matched to three individuals in the stroke group.

Table E2. Baseline characteristics in the matched sample

Baseline characteristics in the matched sample			*p*-value[a]
	**Stroke diagnosis**	**No diagnosis of stroke**	
	N=6,894	N=6,759	
**DEMOGRAPHICS**			
Age at referral - Mean (SD)	57.8 (14.3)	57.8 (14.6)	0.93
	**% (n)**	**% (n)**	
**Age category**			0.45
18-24	1.6 (107)	1.9 (126)	
25-44	15.1 (1,038)	15.5 (1,047)	
45-64	50.8 (3,503)	50.2 (3,392)	
65+	32.6 (2,246)	32.5 (2,194)	
**Ethnicity**			0.85
White	82.3 (5,676)	83.1 (5,616)	
Mixed	1.1 (75)	1.0 (69)	
Asian	3.6 (246)	3.4 (229)	
Black	2.7 (183)	2.5 (171)	
Other	0.9 (63)	0.8 (52)	
Missing	9.4 (651)	9.2 (622)	
**Gender**			0.85
Male	47.9 (3,299)	47.9 (3,240)	
Female	51.8 (3,573)	51.8 (3,501)	
Missing	0.3 (22)	0.3 (18)	
**IMD quintile**			0.48
1 (Most deprived)	21.7 (1,494)	21.7 (1,469)	
2	20.7 (1,426)	19.8 (1,337)	
3	20.0 (1,382)	20.9 (1,411)	
4	18.0 (1,238)	17.2 (1,165)	
5 (Least deprived)	17.0 (1,169)	17.6 (1,191)	
Missing	2.7 (185)	2.8 (186)	
**Employment status**			<0.001
Employed	65.2 (4,498)	74.2 (5,012)	
Unemployed	29.4 (2,024)	20.5 (1,388)	
Missing/preferred not to answer	5.4 (372)	5.3 (359)	
**CLINICAL MEASURES PRE-TREATMENT, PRE-EXISTING CONDITIONS AND MEDICATION**			
	**Mean (SD)**	**Mean (SD)**	
Depression symptoms pre-treatment (PHQ-9)	15.8 (5.6)	16.0 (5.6)	0.13
Anxiety symptoms pre-treatment (GAD-7)	13.5 (4.8)	13.5 (4.7)	0.66
Social Functioning (WSAS) pre-treatment, pro-rated	19.7 (10.4)	18.8 (10.0)	<0.001
	**% (n)**	**% (n)**	
**Taking psychotropic medication**			0.93
Yes	49.2 (3,389)	49.1 (3,318)	
No	41.8 (2,883)	41.7 (2,819)	
Missing	9.0 (622)	9.2 (622)	
**Self-reported long-term condition**			0.97
Yes	56.7 (3,907)	56.7 (3,833)	
No	25.7 (1,775)	25.6 (1,730)	
Missing	17.6 (1,212)	17.7 (1,196)	
**TREATMENT FACTORS**			
**Diagnosis category**			0.051
Depression	37.2 (2,566)	36.3 (2,452)	
Anxiety disorders			
Mixed anxiety and depressive disorder	14.7 (1,012)	15.7 (1,059)	

GAD	14.5 (1,002)	14.6 (985)	
OCD	0.5 (37)	1.0 (67)	
PTSD	2.5 (170)	2.2 (152)	
Phobic anxiety & panic	4.7 (324)	4.9 (330)	
Other anxiety disorder	0.3 (24)	0.4 (28)	
Missing	25.5 (1,759)	24.9 (1,686)	
**Year of treatment**			0.91
2012	0.5 (34)	0.6 (38)	
2013	4.9 (337)	5.2 (352)	
2014	11.1 (766)	11.1 (748)	
2015	16.4 (1,134)	16.3 (1,101)	
2016	18.8 (1,295)	19.1 (1,289)	
2017	20.6 (1,422)	21.0 (1,420)	
2018	22.4 (1,543)	21.5 (1,452)	
2019	5.3 (363)	5.3 (359)	
**Reason for ending therapy**			0.045
Completed	60.0 (4,137)	61.2 (4,135)	
Dropout	21.3 (1,469)	19.7 (1,331)	
Service not suitable	0.8 (56)	0.7 (44)	
Declined	2.7 (185)	2.7 (185)	
Referred on	4.0 (279)	3.6 (242)	
Missing	11.1 (768)	12.2 (822)	
	**Mean (SD)**	**Mean (SD)**	
Number of sessions[b]	6.3 (4.3)	6.6 (4.5)	<0.001
Time between referral and assessment (weeks)[b]	3.1 (3.8)	3.1 (4.1)	0.81
Time between assessment and treatment (weeks)[b]	6.8 (7.0)	6.7 (7.1)	0.88

Abbreviations: IMD = Index of multiple deprivation, GAD = Generalised anxiety disorder, OCD=Obsessive compulsive disorder, PTSD=Post traumatic stress disorder, PHQ=Patient health questionnaire, SD=Standard Deviation, HES=Hospital Episode Statistics, LTC=Long term condition

[a]Independent t-tests were used for continuous variables and chi-squared tests were used for categorical variables

[b]To reduce the influence of extreme values, variables were winsorized at the top 99% percentile

Propensity scores for matching were estimated using logistic regression using age group, gender, ethnicity, LTC case, psychotropic medication, IMD decile as categorical covariates and year of first appointment, baseline PHQ-9, baseline GAD-7, waiting times referral to assessment, waiting time assessment to treatment as continuous covariates.

F) Clinical commissioning group categories

Clinical commission groups (CCGs) were renamed to become sub-Integrated Care Board (ICB) locations from 1st July 2022. The table below lists the organisation codes for CCGs by each ICB. In the analyses for this study, multilevel analyses were conducted to investigate clustering effects by each CCG as listed below.

Sub ICB Location (CCG) Name
NHS LANCASHIRE AND SOUTH CUMBRIA ICB - 00Q
NHS LANCASHIRE AND SOUTH CUMBRIA ICB - 00R
NHS LANCASHIRE AND SOUTH CUMBRIA ICB - 00X
NHS LANCASHIRE AND SOUTH CUMBRIA ICB - 01A
NHS LANCASHIRE AND SOUTH CUMBRIA ICB - 01E
NHS LANCASHIRE AND SOUTH CUMBRIA ICB - 01K
NHS LANCASHIRE AND SOUTH CUMBRIA ICB - 02G
NHS LANCASHIRE AND SOUTH CUMBRIA ICB - 02M
NHS SOUTH YORKSHIRE ICB - 02P
NHS SOUTH YORKSHIRE ICB - 02X
NHS SOUTH YORKSHIRE ICB - 03L
NHS SOUTH YORKSHIRE ICB - 03N
NHS HEREFORDSHIRE AND WORCESTERSHIRE ICB - 18C
NHS MID AND SOUTH ESSEX ICB - 06Q
NHS MID AND SOUTH ESSEX ICB - 07G
NHS MID AND SOUTH ESSEX ICB - 99E
NHS MID AND SOUTH ESSEX ICB - 99F
NHS MID AND SOUTH ESSEX ICB - 99G
NHS BEDFORDSHIRE, LUTON AND MILTON KEYNES ICB - M1J4Y
NHS BIRMINGHAM AND SOLIHULL ICB - 15E
NHS NORTH EAST AND NORTH CUMBRIA ICB - 00L
NHS NORTH EAST AND NORTH CUMBRIA ICB - 00N
NHS NORTH EAST AND NORTH CUMBRIA ICB - 00P
NHS NORTH EAST AND NORTH CUMBRIA ICB - 01H
NHS NORTH EAST AND NORTH CUMBRIA ICB - 13T
NHS NORTH EAST AND NORTH CUMBRIA ICB - 16C
NHS NORTH EAST AND NORTH CUMBRIA ICB - 84H
NHS NORTH EAST AND NORTH CUMBRIA ICB - 99C
NHS DERBY AND DERBYSHIRE ICB - 15M
NHS SUFFOLK AND NORTH EAST ESSEX ICB - 06L
NHS SUFFOLK AND NORTH EAST ESSEX ICB - 06T
NHS SUFFOLK AND NORTH EAST ESSEX ICB - 07K
NHS DEVON ICB - 15N
NHS LINCOLNSHIRE ICB - 71E
NHS LEICESTER, LEICESTERSHIRE AND RUTLAND ICB - 03W
NHS LEICESTER, LEICESTERSHIRE AND RUTLAND ICB - 04C
NHS LEICESTER, LEICESTERSHIRE AND RUTLAND ICB - 04V
NHS SOUTH EAST LONDON ICB - 72Q
NHS KENT AND MEDWAY ICB - 91Q
NHS HERTFORDSHIRE AND WEST ESSEX ICB - 06K
NHS HERTFORDSHIRE AND WEST ESSEX ICB - 06N
NHS HERTFORDSHIRE AND WEST ESSEX ICB - 07H
NHS NORTH EAST LONDON ICB - A3A8R
NHS NORTH CENTRAL LONDON ICB - 93C
NHS NORFOLK AND WAVENEY ICB - 26A
NHS STAFFORDSHIRE AND STOKE-ON-TRENT ICB - 04Y

NHS STAFFORDSHIRE AND STOKE-ON-TRENT ICB - 05D
NHS STAFFORDSHIRE AND STOKE-ON-TRENT ICB - 05G
NHS STAFFORDSHIRE AND STOKE-ON-TRENT ICB - 05Q
NHS STAFFORDSHIRE AND STOKE-ON-TRENT ICB - 05V
NHS STAFFORDSHIRE AND STOKE-ON-TRENT ICB - 05W
NHS FRIMLEY ICB - D4U1Y
NHS SUSSEX ICB - 09D
NHS SUSSEX ICB - 70F
NHS SUSSEX ICB - 97R
NHS SHROPSHIRE, TELFORD AND WREKIN ICB - M2L0M
NHS GREATER MANCHESTER ICB - 00T
NHS GREATER MANCHESTER ICB - 00V
NHS GREATER MANCHESTER ICB - 00Y
NHS GREATER MANCHESTER ICB - 01D
NHS GREATER MANCHESTER ICB - 01G
NHS GREATER MANCHESTER ICB - 01W
NHS GREATER MANCHESTER ICB - 01Y
NHS GREATER MANCHESTER ICB - 02A
NHS GREATER MANCHESTER ICB - 02H
NHS GREATER MANCHESTER ICB - 14L
NHS HUMBER AND NORTH YORKSHIRE ICB - 02Y
NHS HUMBER AND NORTH YORKSHIRE ICB - 03F
NHS HUMBER AND NORTH YORKSHIRE ICB - 03H
NHS HUMBER AND NORTH YORKSHIRE ICB - 03K
NHS HUMBER AND NORTH YORKSHIRE ICB - 03Q
NHS HUMBER AND NORTH YORKSHIRE ICB - 42D
NHS BATH AND NORTH EAST SOMERSET, SWINDON AND WILTSHIRE ICB - 92G
NHS NORTHAMPTONSHIRE ICB - 78H
NHS GLOUCESTERSHIRE ICB - 11M
NHS HAMPSHIRE AND ISLE OF WIGHT ICB - 10R
NHS HAMPSHIRE AND ISLE OF WIGHT ICB - D9Y0V
NHS NORTH WEST LONDON ICB - W2U3Z
NHS SOMERSET ICB - 11X
NHS NOTTINGHAM AND NOTTINGHAMSHIRE ICB - 02Q
NHS NOTTINGHAM AND NOTTINGHAMSHIRE ICB - 52R
NHS CORNWALL AND THE ISLES OF SCILLY ICB - 11N
NHS BUCKINGHAMSHIRE, OXFORDSHIRE AND BERKSHIRE WEST ICB - 10Q
NHS BUCKINGHAMSHIRE, OXFORDSHIRE AND BERKSHIRE WEST ICB - 14Y
NHS BUCKINGHAMSHIRE, OXFORDSHIRE AND BERKSHIRE WEST ICB - 15A
NHS BLACK COUNTRY ICB - D2P2L
NHS CAMBRIDGESHIRE AND PETERBOROUGH ICB - 06H
NHS BRISTOL, NORTH SOMERSET AND SOUTH GLOUCESTERSHIRE ICB - 15C
NHS DORSET ICB - 11J
NHS SOUTH WEST LONDON ICB - 36L
NHS WEST YORKSHIRE ICB - 02T
NHS WEST YORKSHIRE ICB - 03R
NHS WEST YORKSHIRE ICB - 15F
NHS WEST YORKSHIRE ICB - 36J
NHS WEST YORKSHIRE ICB - X2C4Y
NHS COVENTRY AND WARWICKSHIRE ICB - B2M3M
NHS SURREY HEARTLANDS ICB - 92A
NHS CHESHIRE AND MERSEYSIDE ICB - 01F

NHS CHESHIRE AND MERSEYSIDE ICB - 01J
NHS CHESHIRE AND MERSEYSIDE ICB - 01T
NHS CHESHIRE AND MERSEYSIDE ICB - 01V
NHS CHESHIRE AND MERSEYSIDE ICB - 01X
NHS CHESHIRE AND MERSEYSIDE ICB - 02E
NHS CHESHIRE AND MERSEYSIDE ICB - 12F
NHS CHESHIRE AND MERSEYSIDE ICB - 27D
NHS CHESHIRE AND MERSEYSIDE ICB - 99A

G) Adjusting for the Charlson Comorbidity Index

The Royal Society of Surgeon (RCS) Charlson Score (12) was adapted and used in these analyses. The RCS Co-morbidity Consensus Group developed and validated a co-morbidity score for use with administrative data in the UK. The RCS Charlson Score is designed to be used as a count of the number of co-morbid conditions (0, 1, 2, or 3 if there are three or more co-morbid conditions). The RCS Charlson Score was developed with surgical patients in mind, and validated in patients undergoing elective surgery (note that they represent a highly selected group as surgery is offered only to patients who are deemed fit for the procedure).

For the present study, we adapted the RCS Charlson Score to exclude cerebrovascular disease and hemiplegia or paraplegia from the list of contributing conditions (as in Goldstein et al., 2004 (13)), to avoid over-adjusting for morbidity associated with stroke. Relevant ICD-10 codes recorded prior to the date of assessment at NHS TTad were identified from HES and MHSDS datasets to construct the adapted RCS Charlson Scores.

Table G1. Royal College of Surgeons Charlson Score indicating International Classification of Disease tenth revision codes for 14 disease categories

Disease category	ICD-10 codes
Myocardial infarction	I21, I22, I23, I252
Congestive cardiac failure	I11, I13, I255, I42, I43, I50, I517
Peripheral vascular disease	I70–I73, I770, I771, K551, K558, K559, R02, Z958, Z959
Cerebrovascular disease	G45, G46, I60–I69
Dementia	A810, F00–F03, F051, G30, G31
Chronic pulmonary disease	I26, I27, J40–J45, J46, J47, J60–J67, J684, J701, J703
Rheumatological disease	M05, M06, M09, M120, M315, M32–M36
Liver disease	B18, I85, I864, I982, K70, K71, K721, K729, K76, R162, Z944
Diabetes mellitus	E10–E14
Hemiplegia or paraplegia	G114, G81–G83
Renal disease	I12, I13, N01, N03, N05, N07, N08, N171, N172, N18, N19, N25, Z49, Z940, Z992

Disease category	ICD-10 codes
Any malignancy	C00–C26, C30–C34, C37–C41, C43, C45–C58, C60–C76, C80–C85, C88, C90–C97
Metastatic solid tumour	C77–C79
AIDS/HIV infection	B20–B24

AIDS, acquired immune deficiency syndrome; HIV, human immunodeficiency virus

H) Secondary outcome measures

Outcome	Questionnaire measure	Information on measurement
Depression	Patient Health Questionnaire 9-item (PHQ-9) (14)	To measure symptoms of depression, scores of 10 or above indicate clinical caseness for depression, and a reduction of 6 or more points is used to indicate reliable improvement. (15) The PHQ-9 has been validated in screening for post-stroke depression previously. (16)
Anxiety	The Generalized Anxiety Disorder Scale 7-item version (GAD-7) (17)	To assess generalized anxiety symptoms, a cut-off of 8 or higher is used for caseness and 4 or more for reliable improvement. Alternative “anxiety disorder specific measures” (ADSMs) are used when specific anxiety disorders are identified as the “problem descriptor” (18), for example the Social Phobia Inventory (10) for use when social anxiety disorder is identified. When present, these ADSMs are used to calculate NHS TTad outcomes instead of the GAD-7. The full list of ADSMs alongside the service thresholds for caseness and reliable change is presented in Appendix C.
Personal functioning	The Work and Social Adjustment Scale (WSAS) (19)	Measures personal functioning in relation to: ‘ability to work’, ‘home management’, ‘social activities’, ‘private leisure activities’ and ‘close relationships’ (domain score range, 0-8). The WSAS item on the ‘ability to work’ is routinely recorded as “not applicable” for individuals not in employment, as was the case for 33% of adults in the present study sample. For these individuals, their total WSAS score was ‘pro-rated’, meaning the average value of the scores for all other items was imputed as the score for the item on ability to work.

1. National Collaborating Center for Mental Health. The NHS Talking Therapies manual2023. Available from: https://www.england.nhs.uk/publication/the-improving-access-to-psychological-therapies-manual/.
2. NHS Digital. Hospital Episode Statistics (HES)2023 Accessed 2 June 2023. Available from: https://digital.nhs.uk/data-and-information/data-tools-and-services/data-services/hospital-episode-statistics.
3. NHS Digital. Mental Health Services Data Set (MHSDS)2023 Accessed 2 June 2023. Available from: https://digital.nhs.uk/data-and-information/data-collections-and-data-sets/data-sets/mental-health-services-data-set.
4. NHS Digital. Linked HES-ONS mortality data2023 Accessed 2 June 2023. Available from: https://digital.nhs.uk/data-and-information/data-tools-and-services/data-services/linked-hes-ons-mortality-data.
5. Chambless DL, Caputo GC, Jasin SE, Gracely EJ, Williams C. The Mobility Inventory for Agoraphobia. Behaviour Research and Therapy. 1985;23(1):35-44.
6. Salkovskis PM, Rimes KA, Warwick HMC, Clark DM. The Health Anxiety Inventory: development and validation of scales for the measurement of health anxiety and hypochondriasis. Psychological Medicine. 2002;32(5):843-53.
7. Foa EB, Kozak MJ, Salkovskis PM, Coles ME, Amir N. The validation of a new obsessive–compulsive disorder scale: The Obsessive–Compulsive Inventory. Psychological Assessment. 1998;10:206-14.
8. Shear MK, Rucci P, Williams J, Frank E, Grochocinski V, Vander Bilt J, et al. Reliability and validity of the Panic Disorder Severity Scale: replication and extension. Journal of Psychiatric Research. 2001;35(5):293-6.
9. Creamer M, Bell R, Failla S. Psychometric properties of the Impact of Event Scale—Revised. Behaviour Research and Therapy. 2003;41(12):1489-96.
10. Connor KM, Davidson JRT, Churchill LE, Sherwood A, Weisler RH, Foa E. Psychometric properties of the Social Phobia Inventory (SPIN): New self-rating scale. The British Journal of Psychiatry. 2000;176(4):379-86.
11. Leuven E, Sianesi B. PSMATCH2: Stata module to perform full Mahalanobis and propensity score matching, common support graphing, and covariate imbalance testing. Statistical Software Components, Boston College Department of Economics; 2003.
12. Armitage JN, van der Meulen JH, Royal College of Surgeons Co-morbidity Consensus G. Identifying co-morbidity in surgical patients using administrative data with the Royal College of Surgeons Charlson Score. Br J Surg. 2010;97(5):772-81.
13. Goldstein LB, Samsa GP, Matchar DB, Horner RD. Charlson Index comorbidity adjustment for ischemic stroke outcome studies. Stroke. 2004;35(8):1941-5.
14. Kroenke K, Spitzer RL, Williams JB. The PHQ-9: validity of a brief depression severity measure. J Gen Intern Med. 2001;16(9):606-13.
15. NHS Digital. Psychological Therapies: Annual Report on the use of IAPT services - England, 2015-162016 Accessed 4 June 2023. Available from: https://digital.nhs.uk/data-and-information/publications/statistical/psychological-therapies-annual-reports-on-the-use-of-iapt-services/annual-report-2015-16.
16. Williams LS, Brizendine EJ, Plue L, Bakas T, Tu W, Hendrie H, et al. Performance of the PHQ-9 as a screening tool for depression after stroke. Stroke. 2005;36(3):635-8.
17. Spitzer RL, Kroenke K, Williams JB, Lowe B. A brief measure for assessing generalized anxiety disorder: the GAD-7. Arch Intern Med. 2006;166(10):1092-7.
18. Clark DM. Realizing the Mass Public Benefit of Evidence-Based Psychological Therapies: The IAPT Program. Annu Rev Clin Psychol. 2018;14:159-83.
19. Mundt JC, Marks IM, Shear MK, Greist JH. The Work and Social Adjustment Scale: a simple measure of impairment in functioning. Br J Psychiatry. 2002;180:461-4.
