# Supplementary material for: A record-linkage study of post-stroke primary care psychological therapy effectiveness in England
Source: Nat Ment Health. 2025 Jun 5;3(6):626–35. doi: 10.1038/s44220-025-00429-z (PMC12165844; doi:10.1038/s44220-025-00429-z)
Supplement: Supplementary file 2 — Reporting Summary [file 44220_2025_429_MOESM2_ESM.pdf]

nature portfolio

Corresponding author(s): Jae Won Suh

Last updated by author(s): Mar 24, 2025

Reporting Summary

Nature Portfolio wishes to improve the reproducibility of the work that we publish. This form provides structure for consistency and transparency in reporting. For further information on Nature Portfolio policies, see our Editorial Policies and the Editorial Policy Checklist.

Statistics

For all statistical analyses, confirm that the following items are present in the figure legend, table legend, main text, or Methods section.

n/a	Confirmed	
☐	☒	The exact sample size (*n*) for each experimental group/condition, given as a discrete number and unit of measurement
☐	☒	A statement on whether measurements were taken from distinct samples or whether the same sample was measured repeatedly
☐	☒	The statistical test(s) used AND whether they are one- or two-sided<br>*Only common tests should be described solely by name; describe more complex techniques in the Methods section.*
☐	☒	A description of all covariates tested
☐	☒	A description of any assumptions or corrections, such as tests of normality and adjustment for multiple comparisons
☐	☒	A full description of the statistical parameters including central tendency (e.g. means) or other basic estimates (e.g. regression coefficient) AND variation (e.g. standard deviation) or associated estimates of uncertainty (e.g. confidence intervals)
☐	☒	For null hypothesis testing, the test statistic (e.g. *F*, *t*, *r*) with confidence intervals, effect sizes, degrees of freedom and *P* value noted<br>*Give P values as exact values whenever suitable.*
☒	☐	For Bayesian analysis, information on the choice of priors and Markov chain Monte Carlo settings
☒	☐	For hierarchical and complex designs, identification of the appropriate level for tests and full reporting of outcomes
☐	☒	Estimates of effect sizes (e.g. Cohen's *d*, Pearson's *r*), indicating how they were calculated

Our web collection on statistics for biologists contains articles on many of the points above.

Software and code

Policy information about availability of computer code

Data collection	Data collection was not conducted by the authors.
Data analysis	All analyses were conducted in Stata 17. The code used for statistical analyses in this study is publicly available on GitHub at https://github.com/jae-suh74/MODIFY_Stroke.

For manuscripts utilizing custom algorithms or software that are central to the research but not yet described in published literature, software must be made available to editors and reviewers. We strongly encourage code deposition in a community repository (e.g. GitHub). See the Nature Portfolio guidelines for submitting code & software for further information.

Data

Policy information about availability of data

All manuscripts must include a data availability statement. This statement should provide the following information, where applicable:

- Accession codes, unique identifiers, or web links for publicly available datasets
- A description of any restrictions on data availability
- For clinical datasets or third party data, please ensure that the statement adheres to our policy

This work uses data provided by patients and collected by the NHS as part of their care and support. The dataset for the current analyses was provided as part of the MODIFY study. All data used for this study are available upon successful application to NHS Digital via the Data Access Request Service (DARS): https://digital.nhs.uk/ services/data-access-request-service-dars. Data fields can be accessed via NHS Digital data dictionary: https://www.datadictionary.nhs.uk/. Further

information on the datasets are available from the following weblinks: https://digital.nhs.uk/data-and-information/data-collections-and-data-sets/data-sets/improving-access-to-psychological-therapies-data-set; https://digital.nhs.uk/services/hospital-episode-statistics; https://digital.nhs.uk/data-and-information/data-collections-and-data-sets/data-sets/mental-health-services-data-set; https://digital.nhs.uk/services/data-access-request-service-dars/dars-products-and-services/data-set-catalogue/civil-registrations-of-death

Research involving human participants, their data, or biological material

Policy information about studies with human participants or human data. See also policy information about sex, gender (identity/presentation), and sexual orientation and race, ethnicity and racism.

Reporting on sex and gender	Self-reported demographic information from NHS TTad data included gender, categorized into Male or Female. Gender was a key variable reported in the descriptive analyses in this study. Gender was also adjusted for as a covariate in relevant regression models, and included in the estimation of propensity scores when matching adults with a stroke diagnosis with control participants without a stroke diagnosis.
Reporting on race, ethnicity, or other socially relevant groupings	Self-reported demographic information from NHS TTad data included ethnicity. Ethnicity was self-reported at the point of referral using two linked lists of options. The first includes higher-order categories (e.g. Asian or Asian British, Black or Black British, Mixed ethnicity, White, Other ethnic groups) and a second list of sub-categories (e.g. Asian – Indian, Asian – Pakistani, Asian – Bangladeshi, Any other Asian background). In our study, ethnicity was categorized into 'White', 'Mixed', 'Asian', 'Black', and 'Other' in accordance with the Office of National Statistics (ONS)'s higher-order categorization list used in UK Census. Ethnicity was adjusted for in relevant regression models and included in the estimation of propensity scores for matching.
Population characteristics	A cohort was formed of 7,597 adults who had a stroke diagnosis prior to attending NHS TTad, and 1.9 million who did not. Compared with adults without a stroke diagnosis, adults who had a stroke were: substantially older at referral to psychological treatment (mean age 57.8 vs 40.3 years); were more likely to be male; had a lower average Generalized Anxiety Disorder Scale 7-item (GAD-7) score at assessment and depression was more likely to be their presenting complaint (37.7% vs 28.8%). There was no difference in mean Patient Health Questionnaire 9-item (PHQ-9) scores between the two groups at assessment. Those with a stroke diagnosis were also more likely to report taking psychotropic medication and having at least one long-term physical health condition.
Recruitment	Patients were not specifically recruited for this study.
Ethics oversight	All data sources were fully anonymised, and a linkage key was provided by NHS Digital, for records from each database to be linked at the individual patient level using an anonymised subject identifier. Non-identifiable information was provided by NHS Digital with a legal basis for the anonymization, meaning this research did not require research ethics committee review, as per the Governance Arrangements of Research Ethics Committees (GAfREC).

Note that full information on the approval of the study protocol must also be provided in the manuscript.

Field-specific reporting

Please select the one below that is the best fit for your research. If you are not sure, read the appropriate sections before making your selection.

☐ Life sciences ☒ Behavioural & social sciences ☐ Ecological, evolutionary & environmental sciences

For a reference copy of the document with all sections, see nature.com/documents/nr-reporting-summary-flat.pdf

Behavioural & social sciences study design

All studies must disclose on these points even when the disclosure is negative.

Study description	This was a retrospective cohort study of patients who completed a course of treatment via National Health Service (NHS) Talking Therapies for anxiety and depression (TTad), a nationwide primary care psychological therapy service in England, between 2012-2019, whose electronic health records were linked to other national data including hospital episode statistics (HES) and the Mental Health Services Data Set (MHSDS). Patients with a diagnosis of stroke were identified via data linkage; diagnosis of stroke prior to the date of assessment at NHS TTad was identified using ICD-10 (the International Classification of Diseases, 10th Revision) codes I60-I64 in the HES and MHSDS databases. Two major stroke subtypes were identified separately with the following codes: I61 (intracerebral haemorrhage) and I63 (ischaemic stroke). The objective of the study was to investigate the effectiveness of routinely provided psychological therapies in stroke survivors with depression or anxiety, and to compare their treatment outcomes with those of a statistically matched group of patients without a diagnosis of stroke.
Research sample	Data from all NHS Talking Therapies, for anxiety and depression (NHS TTad) services across all clinical commissioning group (CCG) areas in England in 2012-2019 were available, representing all patients referred to the services nationally during that time. Only patients who attended at least two treatment sessions were included in this study, because otherwise, pre-post treatment outcomes cannot be calculated. Exclusions were applied as per the 'Data Exclusions' section below. In total, 1,939,007 patients were included in the analyses, of whom 7,597 (0.4%) had a diagnosis of stroke prior to being assessed through NHS TTad (mean [SD] age at referral 57.8 [14.3] years; 3,956 [52.1%] female) and 1,931,410 without a stroke diagnosis (mean [SD] age at referral 40.3 [14.7] years; 1,292,057 [66.9%] female).

Sampling strategy	Not applicable. This study used data from linked electronic health records which were already collected. The researchers did not recruit any participants or collect new data.
Data collection	This study used data from linked electronic health records which were already collected administrative and clinical purposes. The researchers did not recruit any participants or collect new data.<br><br>In NHS TTad services, a standardized dataset including a range of patient-reported sociodemographic and clinical characteristics is collected at assessment across services, and measures of anxiety and depression symptoms are collected at each clinical contact, as per national mandates. Using a unique patient identification key provided by NHS Digital, each participant was linked across routinely-collected NHS datasets: NHS TTad, HES (including inpatient and outpatient records and associated diagnostic codes), MHSDS, and HES-ONS (Office of National Statistics) mortality data.
Timing	The dataset included all adults aged ≥18 years who were referred to NHS TTad between 2012-2019 and had a linked record in HES, MHSDS, or HES-ONS. The exact dates of referral to NHS TTad were between 1 April 2012 to 29 March 2019.
Data exclusions	If participants had more than one episode of treatment in an NHS TTad service, only data from the first episode were used. A standard set of criteria used in analyses of outcomes in NHS TTad samples were used to exclude those who: 1) had fewer than two sessions of psychological therapy) did not meet the clinical threshold for depression (scored <10 on Patient Health Questionnaire 9-item [PHQ-9]), or generalized anxiety disorder (scored <8 on Generalized Anxiety Disorder Scale 7-item [GAD-7]), or relevant Anxiety Disorder Specific Measure (ADSM) (see Appendix B), 3) had a primary diagnosis for which there is no evidence-based psychological therapy offered in NHS TTad (such as schizophrenia, bipolar disorder, alcohol dependency, bereavement), 4) were still undergoing treatment in the available episode records. Patients missing data on baseline or follow-up measures on the PHQ-9 or GAD-7 were also excluded, but only accounted for <1.5% of the sample who received two or more sessions of treatment. For this study, participants who had a record of stroke during or after their first NHS TTad treatment were excluded. See Appendix C for study flowchart.
Non-participation	Not applicable. This study used data from linked electronic health records which were already collected. The researchers did not recruit any participants or collect new data.
Randomization	Not applicable. Randomization was not relevant to this observational study, as it did not involve experimental conditions or the assignment of patients to different groups or treatments.

Reporting for specific materials, systems and methods

We require information from authors about some types of materials, experimental systems and methods used in many studies. Here, indicate whether each material, system or method listed is relevant to your study. If you are not sure if a list item applies to your research, read the appropriate section before selecting a response.

Materials & experimental systems

n/a	Involved in the study	
☒	☐	Antibodies
☒	☐	Eukaryotic cell lines
☒	☐	Palaeontology and archaeology
☒	☐	Animals and other organisms
☒	☐	Clinical data
☒	☐	Dual use research of concern
☒	☐	Plants

Methods

n/a	Involved in the study	
☒	☐	ChIP-seq
☒	☐	Flow cytometry
☒	☐	MRI-based neuroimaging

Plants

Seed stocks	n/a
Novel plant genotypes	n/a
Authentication	n/a
